# Circulation and colonisation of *Blastocystis* subtypes in schoolchildren of various ethnicities in rural northern Thailand

**DOI:** 10.1017/S0950268823000596

**Published:** 2023-04-27

**Authors:** Abby McCain, Lucsame Gruneck, Siam Popluechai, Anastasios D. Tsaousis, Eleni Gentekaki

**Affiliations:** 1 School of Science, Mae Fah Luang University, Chiang Rai, Thailand; 2 Gut Microbiome Research Group, Mae Fah Luang University, Chiang Rai, Thailand; 3 Laboratory of Molecular and Evolutionary Parasitology, RAPID Group, School of Biosciences, University of Kent, Canterbury, UK

**Keywords:** *Blastocystis*, BMI, children, colonisation, One Health, subtyping

## Abstract

*Blastocystis* is a protist of controversial pathogenicity inhabiting the gut of humans and other animals. Despite a century of intense study, understanding of the epidemiology of *Blastocystis* remains fragmentary. Here, we aimed to explore its prevalence, stability of colonisation and association with various factors in a rural elementary school in northern Thailand. One hundred and forty faecal samples were collected from 104 children at two time points (tp) 105 days apart. For tp2, samples were also obtained from 15 animals residing on campus and seven water locations. Prevalence in children was 67% at tp1 and 89% at tp2, 63% in chickens, 86% in pigs, and 57% in water. Ten STs were identified, two of which were shared between humans and animals, one between animals and water, and three between humans and water. Eighteen children (out of 36) carried the same ST over both time points, indicating stable colonisation. Presence of *Blastocystis* (or ST) was not associated with body mass index, ethnicity, birth delivery mode, or milk source as an infant. This study advances understanding of *Blastocystis* prevalence in an understudied age group, the role of the environment in transmission, and the ability of specific STs to stably colonise children.

## Introduction

*Blastocystis* is a unicellular eukaryote inhabiting the gastrointestinal tract of vertebrates [1–3] and invertebrates [4, 5]. The organism comprises the most common protist identified in human stool samples [6]. The genetic heterogeneity of *Blastocystis* is extremely high based on the small subunit ribosomal RNA (SSU rRNA) gene [7–14]. To date, 34 subtypes (STs; ST1–ST17, ST21, and ST23–ST38) have been identified in avian and mammalian hosts. Of these, 14 have been recorded in humans (ST1–ST10, ST12, ST14, ST16, and ST23) [13, 15–20], with ST1–ST3 accounting for the majority of human carriage [21, 22]. The remaining STs mostly colonise non-human endothermic hosts [1, 6].

The pathogenicity of *Blastocystis* is a topic of ongoing debate. Its presence in the gut was initially linked to gastrointestinal symptoms, primarily due to a lack of information regarding carriage in healthy populations. The recent burst of studies reporting *Blastocystis* in individuals with no gastrointestinal symptoms has brought forth the hypothesis that the organism is a common member of the human gut microbiota [23, 24]. To that end, low prevalence of *Blastocystis* has been negatively associated with irritable bowel syndrome (IBS) [25]. However, positive associations between IBS patients and the presence of *Blastocystis* have also been observed. These conflicting results reinforce its controversial pathogenicity status [26–31]. Links between *Blastocystis* STs and/or strains and pathogenicity have also been proposed, but not conclusively shown [32–34].

*Blastocystis* has a worldwide distribution, having been found in both industrialised and non-industrialised countries [35–39]. ST3 is the most commonly distributed globally, followed by ST1 and ST2 [21, 22]. Several factors have been looked at in association with the presence or absence of *Blastocystis.* For instance, prevalence in rural communities is typically higher than that in urban areas [40, 41]. The age of the host seems to also play a role in colonisation, with carriage being generally higher in children [42–45].

The prevalence of *Blastocystis* in children varies, ranging from 4% [46] to 100% [36]. The majority of studies in this age group are microscopy-based; hence, subtyping information is relatively sparse [46–48]. In these studies, ST1–ST3 were the most commonly detected, following the global trend [49–52]. ST6 and ST7 have also been identified, most prominently in low- and middle-income countries (LMICs), but with lower carriage [44, 53–55]. Recently, the first occurrence of ST10, ST14, and ST16 was reported in children [16, 17]. The presence of *Blastocystis* in children was linked to sanitary habits, water source/treatment, type of housing, and socioeconomic status of parents [48, 56, 57]. Most subtyping studies based on children have originated in South America [17, 54, 58], with only comparatively few from Asia. Given these gaps, studies addressing both the presence and STs of *Blastocystis* in children are needed worldwide.

In this study, we collected faecal samples from children of different ethnicities with no gastrointestinal symptoms in grades 1–6 attending a rural school in the Chiang Rai province, northern Thailand. The prevalence and genetic diversity of *Blastocystis* were determined. Information on body mass index (BMI), ethnicity, age, birth delivery mode, and milk source as an infant was also collected and assessed for association with *Blastocystis* presence. Faecal samples from animals raised on the school grounds and environmental samples were also examined to assess the circulation of the organism.

## Methods

### Ethics

The Ethics committee of Mae Fah Luang University approved the collection of human faecal samples (Ethics Registry: EC19359-11). The process, conditions, and ethical rules are in compliance with the Declaration of Helsinki. Data were strictly anonymised with each sample assigned an individual barcode. The parents and/or legal guardians of all participants provided a signed informed consent form.

### Study area and sample collection

**Figure 1.**
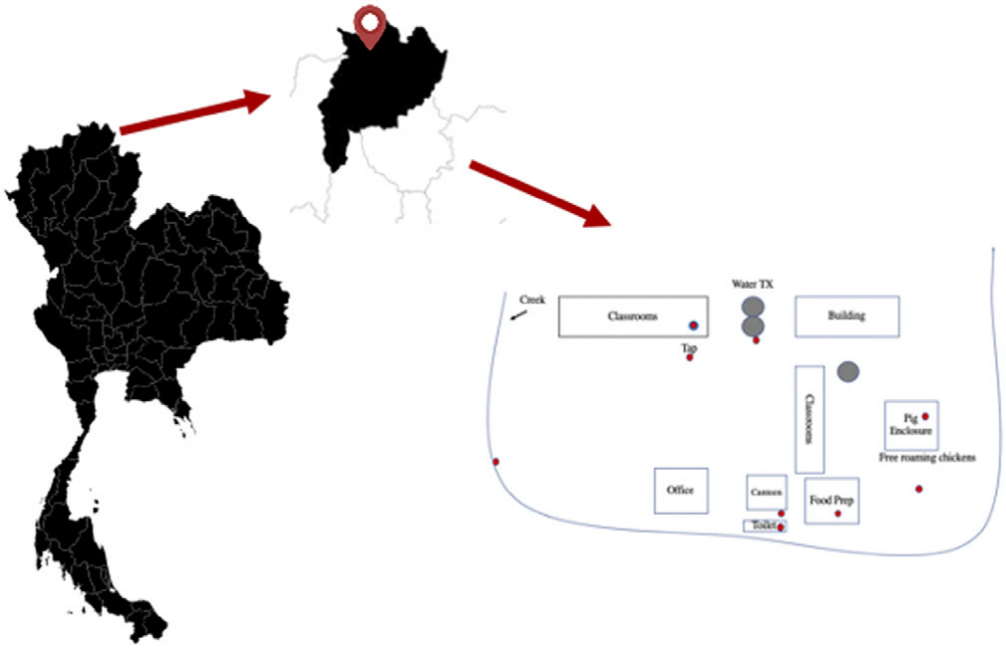
Left panel: Map of Thailand. Middle panel: Close-up of Chiang Rai Province. Right panel: Details of the school grounds. Red dots indicate collection sites.

The present study took place in a rural area in the northern part of Thailand (Figure 1). Students enrolled in grades 1–6 (6–14 years old) at an elementary school in the Mae Fah Luang district of Chiang Rai province were recruited for the study. Recruitment of students took place via voluntary participation. The principal, staff, and parents and/or legal guardians were invited to attend a meeting, during which the objectives of the study were conveyed in detail in the local language. Proper sterile technique in the collection of stool samples was also emphasised. Time was set aside for discussion, during which both children and adults were given the opportunity to ask questions and/or clarifications about any part of the project. A total of 104 volunteers participated. The participants were Akha (*n* = 29), Burmese (*n* = 1), Chinese (*n* = 25), Lahu (*n* = 5), Thai (*n* = 19), and Thai Yai (*n* = 25).

Samples were collected in November 2019 [time point 1 (tp1)] and February 2020 [time point 2 (tp2)] over the course of several days each time. Prior to collection, students were provided with a sterile container labelled with a unique ID code, ensuring confidentiality. On the morning of collection, one sample from each child was obtained at their homes and in some cases at the school. Samples were immediately stored at 4°C. Containers containing stool were collected from the students upon arrival to school and placed on ice until transport to the laboratory, where they were stored at −80°C. Information on ethnicity, age, sex, childbirth delivery mode (normal birth vs. Caesarean section), and mode of milk feeding (breast milk, formula, and mix of both) as an infant was recorded from questionnaires distributed to students (Supplementary Table S1). The questionnaires were filled in by the parents with the help of the school principal. Body weight and height information was also obtained at this time and used to calculate BMI. The BMI was converted into gender-specific *z*-scores for BMI-for-age according to BMI cut-offs for children (5–19 years) set by the World Health Organization. z-scores for BMI-for-age were classified into five groups: severely underweight (<−3 SD; *n* = 1), underweight (≥−3 SD to <−2 SD; *n* = 4), healthy (≥−2 SD to ≤1 SD; *n* = 65), overweight (OV; >1 SD to ≤2 SD; *n* = 16), and obese (OB; >2 SD; *n* = 17). None of the students had a history of gastrointestinal disease, were presented with diarrhoea episodes, or had taken probiotic supplements one month prior to either collection. Diarrhoea was defined as loose, watery stool three or more times per day. None had been treated with antibiotics two months prior to either sample collection. Students eat lunch at the school canteen and are involved in taking care of the school gardens.

At tp2, faecal samples were also obtained from chickens (*n* = 8) and pigs (*n* = 7). A large dog visits the school grounds regularly; however, it was not possible to collect its stool. The chickens roam freely on school grounds and have been present for several years (Supplementary Figure S1). Chicken stool samples were collected in various areas of the school. The pigs were kept in different cement enclosures based on age (Supplementary Figure S2). Pre-weaned piglets were housed in two different enclosures, totalling over a dozen altogether. Adult pigs were housed in two enclosures (three in one enclosure and two in another). Two faecal samples from each pig enclosure were collected, excluding one adult enclosure where only one sample was obtained. Care was taken to collect the stool at the moment of defecation, and none of the animals were diarrheic. The animal stool was immediately inoculated into tubes containing liquid yeast serum growth media (LYSGM) media [59, 60] and incubated at 37°C for up to 3 days before further analysis.

Water samples (*n* = 7) were obtained at tp2 from seven different locations at the elementary school. Of those, five were collected from different water sources on campus, all flowing from two different origins. Water is supplied from two identical water treatment systems (Supplementary Figure S3). Each system is composed of four cylindrical cement tanks. All tap water available on campus runs from these two water treatment systems via plastic pipes. Samples were collected from taps outside of the bathroom, a water collection container inside the bathroom, a tap in the food preparation area, directly from a water tank, and a tap outside of the classroom (Supplementary Figure S4). One water sample was collected from a water container from which students drink daily, whereas another was obtained from the creek that runs along the campus (Supplementary Figure S5). The creek is approximately 3 metres wide and had a slow steady flow at the site of collection. During the time of collection, the water level was very low (a few centimetres) and the rock bed was above water in the middle of the creek. The collected water was slightly cloudy, as a small amount of sediment was present. The creek is accessible to the chickens roaming the school grounds. Approximately 1.5–3 ml of water was collected using sterile, disposable pipettes. The water was immediately inoculated into LYSGM media to a maximum volume of 15 ml and tubes were incubated at 37°C for up to 3 days.

### DNA extraction

Total genomic DNA was extracted from 200 to 400 mg of human faeces using the QIAamp DNA stool mini kit (QIAGEN Inc., Hilden, Germany) according to the manufacturer’s specifications. DNA was extracted for all 104 faecal samples from tp2, whereas for tp1, 36 samples were randomly selected. DNA from animal and environmental samples was extracted prior to the first passage of culture using 200–400 ml of sediment from each sample. For these samples, the G-spin™ Total DNA Extraction Mini Kit (iNtRON, Seongnam, Korea) was used. The isolated DNA from all the samples was kept at −20°C until analysed.

### Molecular detection and subtyping of Blastocystis sp.

#### Polymerase chain reaction

PCR amplification of the SSU rRNA gene was performed using a nested PCR reaction. In the first reaction, the broadly specific oligonucleotide primers RD5 (5’-GGAAGCTTATCTGGTTGATCCTGCCAGTA-3’) and RD3 (5’-GGGATCCTGATCCTTCCGCAGGTTCACCTAC-3’) were used [61]. The thermocycling profile was as follows: denaturing at 94°C for 3 min; 35 cycles of 100 s at 94°C, 100 s at 65°C, 100 s at 72°C, and a final extension of 10 min at 72°C. In the second reaction, the product from the first PCR reaction was used as the template along with the barcoding primers BsRD5F (5’-ATCTGGTTGATCCTGCCAGT-3’) and BhRDr9R (5’-GAGCTTTTTAACTGCAACAACG-3’) [62]. The thermocycling profile was as follows: denaturing at 94°C for 3 min; 35 cycles of 1 min at 94°C, 1 min at 60°C, 90 s at 72°C, and a final extension of 10 min at 72°C. The final reaction amplified an approximately 600 bp region that is typically used for the subtyping of *Blastocystis* *[* *62* *[* *.* The PCR amplicons of the target size were purified using AccuPrep® Gel Purification Kit (Bioneer Corporation, Daejeon, Republic of Korea) according to the manufacturer’s specifications. Purified products were sent for sequencing to Bionics Co., Ltd., Seoul, South Korea. qPCR was performed on all DNA samples that were negative by conventional PCR following a previously developed protocol [15, 63]. Amplifications were performed using the primers BL18SPPF1 (5’-AGTAGTCATACGCTCGTCTCAAA-3’) and BL18SR2PP (5’-TCTTCGTTACCCGTTACTGC-3’), generating a 320 bp product. All PCR and qPCR products were sequenced unidirectionally using the reverse PCR (BhRDr9) and qPCR (BL18SR2PP) primers, respectively.

#### Cloning

Samples with unclear chromatograms were cloned using pLUG-Prime® TA-cloning Vector Kit II (iNtRON, Seongnam, Korea). A total of 32 samples were cloned. Of these, 25 were qPCR products, whereas 7 were PCR products. Thirty-one of the samples were from humans (3 from tp1 and 28 from tp2), and one was from a water source. With regard to PCR products, two colonies per transformation were screened in four cases, and one colony in the remaining two. For the qPCR products, two colonies per transformation were screened in 13 cases, and one colony in the remaining 15. In total, 47 clones were screened. Forty-five of the clones were from children’s samples, whereas two were from a water sample.

### Phylogeny and sequence analysis

Raw data chromatograms were edited, and ambiguous quality bases were removed from the 5’ and 3’ ends using the software AliView [64]. The sequences were used as queries to perform a BLAST search to check for contamination. A dataset was assembled containing reference sequences from all STs, including reptile and insect lineages. Sequences were aligned using MAFFT version 7 [65]. Ambiguous positions were removed using trimAl version 1.3 (http://phylemon.bioinfo.cipf.es/) [66]. One hundred and seventy-five taxa were present in the alignment, and 1,392 positions were left after trimming. A maximum likelihood phylogeny was inferred using RAxML version 8 on XSEDE available on the CIPRES gateway (http://www.phylo.org/portal2/home.action) [67]. The estimates of the genetic distance between sequences that were identified as the same ST and derived from children and water or children and animal were analysed using Kimura two-parameter model embedded in MEGAX [68–70].

### Statistical analysis

Fisher’s exact test with Monte Carlo simulation was used to determine the associations of BMI *z*-score, ethnicity, delivery mode, and milk source variables with the presence of *Blastocystis.* The associations of these variables with the prevalence of *Blastocystis* STs were also determined using the same test. One sample was excluded from the BMI *z*-score analysis, as weight and height information was not collected. Multiple correspondence analysis (MCA) was performed to explore the relationships between variables and the prevalence of *Blastocystis* STs using FactoMineR version 2.4 [71]. The confidence ellipses around the categories (variables) represented in the MCA plot were plotted using Factoextra version 1.0.7 [72]. Contingency tables were visualised using the function balloon plot (R gplots package version 3.1.1) [73]. All the analyses were performed in R software version 4.0.3 [74].

## Results

### Blastocystis prevalence in faecal and environmental samples

For tp1, 12 human faecal samples were positive for *Blastocystis* using nested PCR and 12 additional ones using qPCR (67%, 24/36). For tp2, 27 human faecal samples were positive for *Blastocystis* using nested PCR. Of these, five were false positives (no significant match by BLAST or plant species). The remaining 82 samples were subsequently screened by qPCR, and 71 of them were positive. Thus, the overall prevalence of *Blastocystis* in children at tp2 was 89% (93/104) (Table 1). Sanger sequencing was used to sequence all the samples that were positive by either PCR or qPCR. The overall prevalence of *Blastocystis* in pigs and chickens was 86% (6/7) and 63% (5/8), respectively. Of the seven water samples, four were positive for *Blastocystis*, giving an overall prevalence of 57%. All the sequences generated in this study were submitted to GenBank under accession numbers QQ571480-QQ571626.

**Table 1.** Prevalence and subtype distribution in children, animal, and water samples

Source	Prevalence (%)	ST1	ST2	ST3	ST5	ST6	ST7	ST15	ST26	UNK/mix
Human	93/104 (89)	7	19	42	1	–	14	–	1	5/2
Chicken	5/8 (63)	–	–	–	–	1	4	–	–	–
Pig	6/7 (86)	–	–	–	5	–	–	1	–	–
Water	4/7 (57)	2	–	1	–	–	–	–	–	0/1
	Total	9	19	43	6	1	18	1	1	5/3

### Subtyping of Blastocystis

A total of 25 sequences (21 PCR/qPCR products + 4 clones) were subtyped at tp1, and in total five STs were identified: ST1 (2/25, 8%), ST2 (3/25, 12%), ST3 (15/25, 60%), ST10 (2/25, 8%), and ST23 (1/25, 4%). A case of ST1 and ST3 co-occurrence was also noted. At tp2, a total of eight STs were identified: ST1–ST3, ST5–ST7, ST15, and ST26 (Table 1). Six out of the eight STs identified at tp2 were found in children: ST3 (*n* = 43), ST2 (*n* = 19), ST7 (*n* = 14), ST1 (*n* = 8), ST5 (*n* = 1), and ST26 (*n* = 1). There were four co-occurrences in the children’s samples: ST3/UNK (*n* = 1), ST7/UNK (*n* = 1), and ST1/ST3 (*n* = 2). Five sequences were designated as unknown. Although it was possible to discern that they belonged to *Blastocystis* due to their short length, an ST could not be confidently assigned. Chickens had ST7 (4/5, 80%) and ST6 (1/5, 20%); pigs had ST5 (5/6, 83%) and ST15 (1/6, 17%). ST1 (2/4, 50%) and ST3 (1/4, 25%) along with one co-occurrence of ST1/ST7 were identified in water samples.

### Stability of Blastocystis colonisation

Of the 36 children for which both time points are available, 12 were negative at tp1 (Table 2). All 12 tp1 negatives were positive at tp2 as follows: one with ST2, four with ST3, and seven with ST7. In six instances, a different ST was detected in the two time points. Eighteen children carried the same ST at both time points as follows: ST1 (*n* = 2), ST2 (*n* = 3), and ST3 (*n* = 13). In seven cases, the sequences between the two time points were identical: ST1 (*n* = 1), ST2 (*n* = 2), and ST3 (*n* = 4). In the remaining 11 cases, the sequences differed between 0.18% and 2.97% (Supplementary Table S2).

**Table 2.** Samples collected at two time points, 105 days apart and the subtypes present

Sample	tp1	tp2
MA203	ST1/ST3	ST3
MA204	–	ST7
MA205	ST2	ST2
MA210	ST3	ST3
MA217	ST3	ST3
MA227	ST3	ST3
MA235	–	ST3
MA236	ST10	ST2
MA241	ST3	ST3
MA246	–	ST7
MA256	–	ST3
MA270	–	ST7
MA272	ST3	ST1
MA280	ST3	ST3
MA282	–	ST7
MA286	ST1	ST1
MA291	ST2	ST2
MA296	ST3	ST3
MA300	–	ST3
MA302	–	ST3
MA303	–	ST7
MA307	ST3	ST2
MA309	ST3	ST3
MA311	ST3	ST3
MA318	–	ST7
MA320	ST3	ST3
MA323	–	ST2
MA325	ST3	ST3
MA327	ST2	ST2
MA331	ST1	ST1
MA332	ST10	ST7
MA334	ST3	ST3
MA336	–	ST7
MA341	ST3	ST1
MA344	ST3	ST3
MA348	ST23	ST7

*Note*: Dash indicates a negative result.

Abbreviation: tp, time point.

### Circulation of Blastocystis subtypes

**Figure 2.**
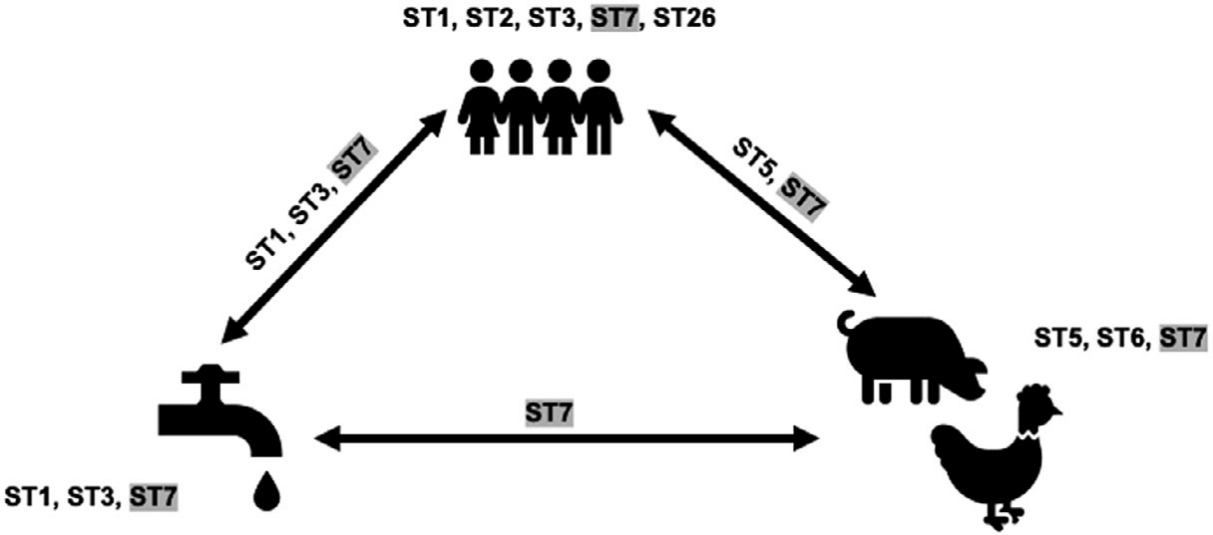
*Blastocystis* subtypes (STs) present in humans, animals, and water in a rural elementary school in northern Thailand. STs on arrows indicate overlap. Shaded STs indicate the presence in all sources considered in this study.

This analysis involved only sequences from tp2. ST sharing was noted among all three categories herein: humans, animals, and water (Figure 2). ST5 and ST7 were shared between humans and animals, ST1, ST3, and ST7 between humans and water, and ST7 between animals and water. ST7 was shared by all three. The ST5 sequences from humans and pigs were not identical. Similarly, ST1 sequences from water and children differed. ST3 sequences from the water creek, and 16 children were identical. ST7 sequences from two children samples and a water sample obtained from the tap outside the school bathroom were also identical.

### Phylogenetic analysis

**Figure 3.**
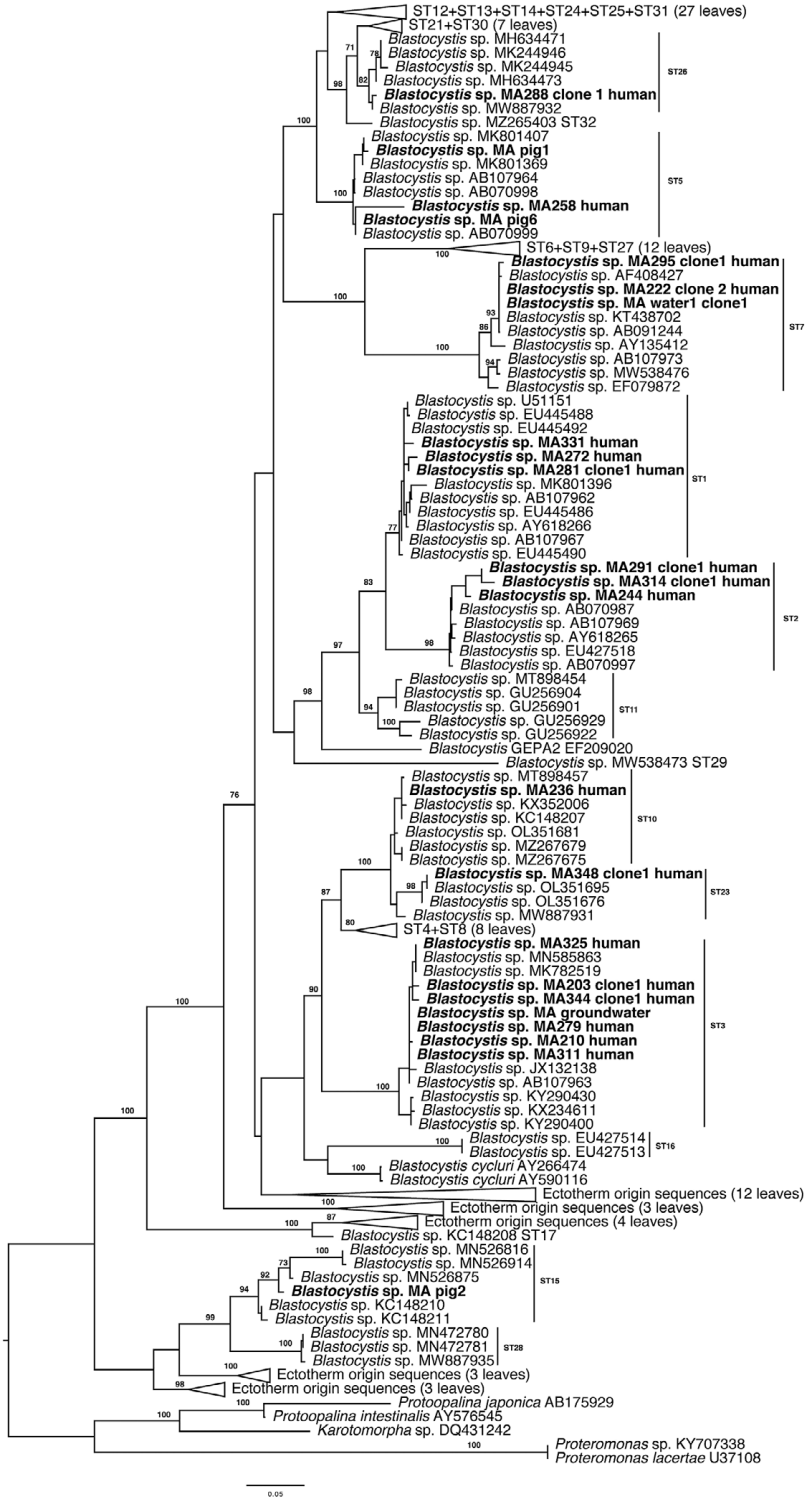
Maximum likelihood phylogeny inferred from 175 *Blastocystis* sequences and 1,392 sites of the *SSU* rRNA gene. Newly generated sequences are in bold font. Numerical values indicate bootstrap support. Only values above 70 are shown.

For the phylogenetic analysis, 23 sequences were chosen to represent the different STs present in the sample cohort (Figure 3). ST15 found in the pig sample grouped with previously published ST15 sequences and together with ST28 and sequences from ectothermic hosts placed at the base of the tree. Newly generated sequences placed within clades consisting of known STs. Sequences identified as ST10 and ST23 were grouped with similar sequences from our previous One Health study [15].

### Association of *Blastocystis* with BMI, ethnicity, birth delivery mode, and milk source

For this analysis, only samples from tp2 were included. The BMI data of two participants were excluded. In the case of one participant, the data were not sufficient to calculate BMI, whereas another was underweight. As there was only one participant in the underweight category, it was not included in the BMI analysis calculations. A graph of contingency tables was constructed, and Fisher’s exact tests were performed.

**Figure 4.**
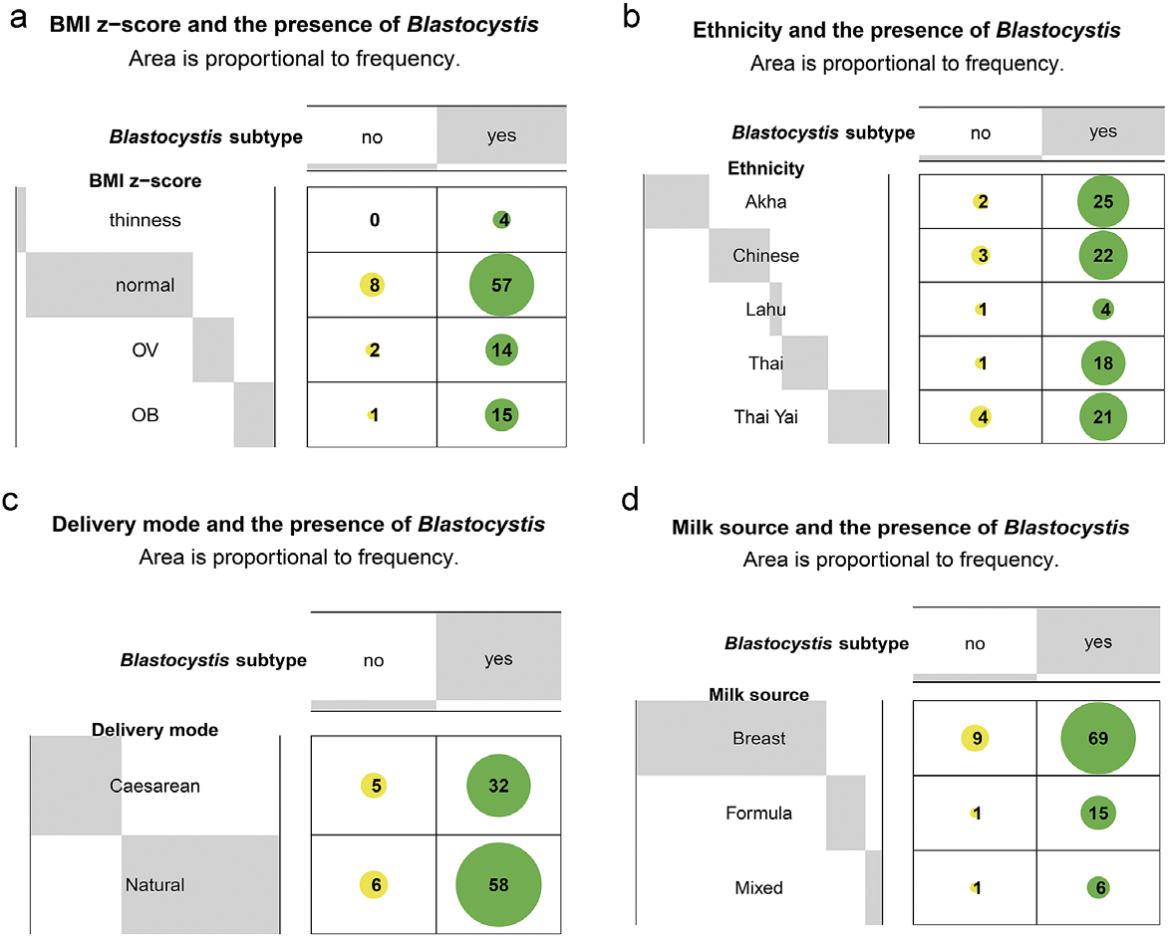
Balloon plots of contingency tables of the relationship between variables (rows) and the presence of *Blastocystis* (columns) in 102 samples. Values represent the frequencies of the presence/absence of *Blastocystis.* (a) Body mass index *z*-score. (b) Ethnicity. (c) Birth delivery mode. (d) Milk source.

**Figure 5.**
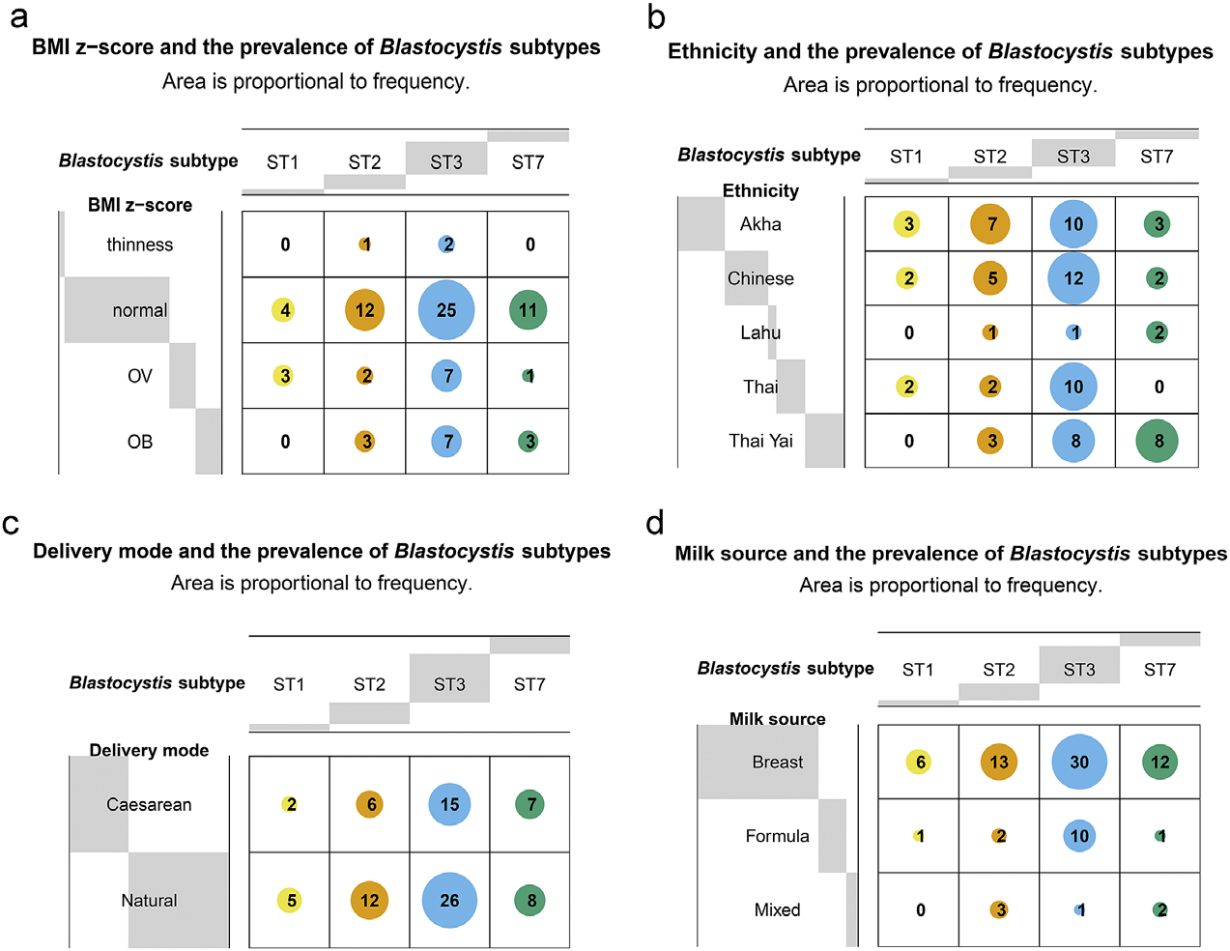
Balloon plots of contingency tables of the relationship between variables (rows) and the prevalence of *Blastocystis* subtypes (STs; columns) in 81 samples. Values are the frequencies of *Blastocystis* STs. (a) Body mass index *z*-score. (b) Ethnicity. (c) Birth delivery mode. (d) Milk source.

At the *Blastocystis* level, there was no significant dependence between its presence and BMI *z*-score, ethnicity, birth delivery mode, or milk source groups (*p* > 0.05) (Figure 4). At the ST level, there were also no significant associations between their prevalence and any of the variables (*p* > 0.05) (Figure 5). The four most abundant STs (ST1–ST3 and ST7) were found across all groups, with two exceptions: ST1 and ST7 were absent in the Thai Yai and Thai ethnicities, respectively.

**Figure 6.**
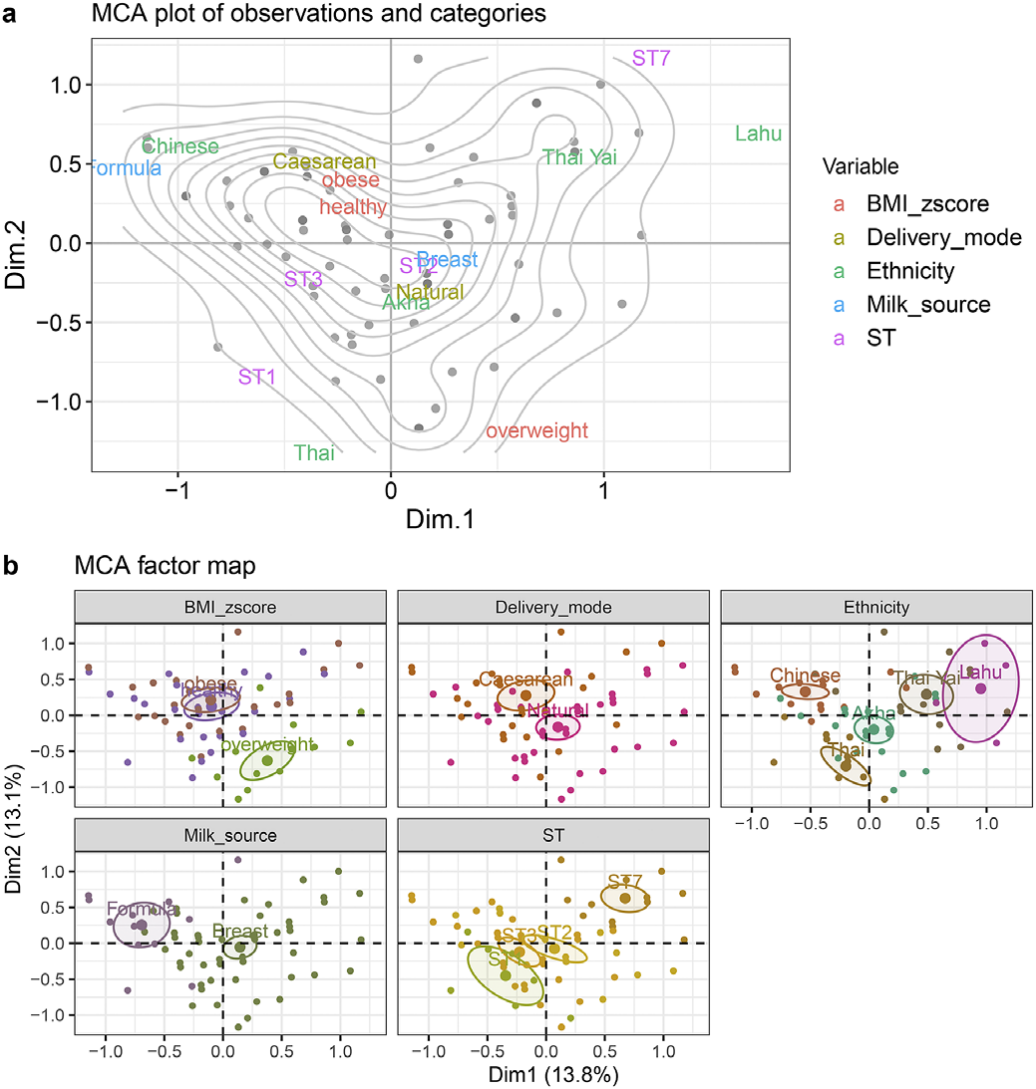
Multiple correspondence analysis (MCA) plots of the association between variables and the prevalence of *Blastocystis* subtypes (*n* = 81). (a) MCA plot displaying the observations and the categories. Density (grey) curves indicate the zones that are highly concentrated. (b) MCA factor map with 95% confidence ellipses surrounding the variables used in this study.

MCA explained 13.8% and 13.1% of individual variability in Dim1 and Dim2, respectively (Figure 6). In Dim1, Lahu ethnicity highly contributed to the dimension (coordinate = 0.80, *p* < 0.001). Breastfeeding (coordinate = 0.42, *p* < 0.0001) and natural birth (coordinate = 0.14, *p* = 0.03) showed a contrasting profile to formula feeding (coordinate = −0.42, *p* < 0.0001) and caesarean (coordinate = −14, *p* = 0.03), respectively. In Dim 2, the variation of observations was mostly characterized by ethnicity (*R* ^2^ = 0.51, *p* < 0.0001), BMI *z*-score (*R* ^2^ = 0.37, *p* < 0.0001), and *Blastocystis* STs (*R* ^2^ = 0.34, *p* < 0.0001), respectively. According to the MCA plots of the observations and categories (Figure 6), there were two density zones clustering around the caesarean, obese, and healthy groups as well as around the natural birth, Akha ethnicity, breastfeeding, and ST2 groups, indicating that the majority of individuals in this study shared a similar pattern among these two clustered groups of variables.

## Discussion

This study took place at an elementary school located in a rural area of northern Thailand. The overall prevalence of *Blastocystis* was 89%, the highest rate reported in children in Thailand to date. Previous studies on this age group in Thailand focused on children from childcare centres and orphanages located in rural or low socioeconomic areas with overall prevalence ranging from 4.8% to 31.9% [50, 57, 75–78]. The variable sensitivity of the methods used for detection across studies likely accounts for the different rates of prevalence. Regardless, the high prevalence rate herein matches previous works on children residing in LMICs in Asia, Africa, and South America [16, 43–45, 55, 79]. The highest carriage ever observed was 100% in children from the Senegal River basin [36].

Identification of *Blastocystis* in children has been primarily microscopy-based with only a limited number of studies focusing on subtyping. In this study, ST3 was the most dominant with a prevalence of 45% followed by ST2 (20%) and ST7 (15%). This pattern does not follow the global trend, where the top three STs are ST1–ST3 [7, 16, 22, 24]. Nonetheless, differences in ST distribution based on geographic location have been observed. For example, in Indonesia and at the China–Myanmar border, ST1, ST3, and ST4 were identified in children, the latter being an ST most commonly observed in European countries [43, 45]. Here, a total of eight STs were identified in children: ST1–ST3, ST5, ST7, ST10, ST23, and ST26. This high ST diversity in terms of number matches previous research on children living in rural areas of South American and African countries [16, 17, 53–55]. In contrast, subtyping work in this age range from southeast Asia has identified remarkably less ST diversity [43, 45, 46, 50, 57, 76]. ST10 and ST23 were previously identified in adults from the same province, but from different districts, suggesting that these are usual, but low-frequency STs in the area. In this study, we also report the first occurrence of ST26 in humans, which has so far been identified only in artiodactyls in the United States, China, and Spain [9, 80, 81]. Even though the sequence is relatively short, both the blast result and the robust phylogenetic placement confirm this identification. The increasing occurrence of these ‘non-human’ STs challenges our previous understanding of the presence of *Blastocysti*s** in humans.

To examine the stability of *Blastocystis* colonisation, we sampled 36 children at two time points 105 days apart. Only three studies have so far explored temporal colonisation, all of which took place in Europe [82–85]. Two of these involved adults, whereas one focused on infants and toddlers. Nevertheless, this is the first study focusing on children (ages 6–14) from an Asian country having a high number of positive samples at both time points. For 18 children, the same STs were detected at both sampling points. These included ST1–ST3, matching the previous temporal colonisation studies, where the same STs along with ST4 and ST8 were found [82–85]. In the rest of the cases herein, the STs were either lost or switched. Notably, even though ST7 was present in seven individuals at the second time point, it was not detected in any of them at the first. This raises the interesting possibility of whether ST7 can stably colonise the human gut or if it is merely a passenger. Alternatively, the observed losses and/or switches could be due to the cycling of STs in the host or double colonisation, whereby only the dominant ST is amplified. Regardless, our data point towards ST1–ST3 being stable colonisers in these children.

In our previous One Health study of *Blastocystis,* we identified similar STs circulating between humans, animals, and the environment and suggested expanding this to additional communities [15]. Hence, here, we collected human and animal stool and environmental samples to explore the circulation of *Blastocystis* and its STs. We found shared STs between hosts and the environment. The zoonotic and waterborne transmission of the various STs has been suggested multiple times [2, 86–92]. Taking this a step further, our genetic distance comparisons identified identical ST3 and ST7 sequences only between children and water. This hints at the waterborne transmission of these STs. A possible explanation for their environmental persistence is a higher resistance to a water environment [93, 94]. Future studies focusing on *Blastocystis* circulation should consider the sequences beyond the ST level.

Fisher’s exact test was used to determine the association between the occurrence of *Blastocystis* and certain variables. The results indicated that there was no association between the presence of *Blastocystis* and BMI *z*-score, ethnicity, birth delivery mode, and milk source. This is likely due to the high prevalence of the organism across all categories in this cohort. To date, there have not been *Blastocystis* studies in children that evaluate any of these factors. BMI has been considered in past investigations involving adults [95, 96]. The presence of *Blastocystis* has been observed to have a strong negative correlation with BMI; specifically, higher prevalence is found in lean individuals [35, 97, 98]. Data from these studies have not shown *Blastocystis* and BMI to be ST-specific [35]. Our statistical analysis suggested that lean children were more likely to be colonised with ST1, whereas obese children were more likely to be colonised with ST3. Further investigations are necessary for the overall microbiome and metabolome [99] level to disentangle relationships between STs and BMI.

This study combines a One Health approach in a rural school community focused on a microbial eukaryote of controversial pathogenicity. Aside from the high prevalence and stability of *Blastocystis* ST1–ST3 colonisation in children, this study raises a few important questions: which STs are colonisers or passengers and what are the determining factors? Do anthropometric factors and ethnicity play a role? What is the environmental contribution in transmission? Are all these factors associated with colonisation at the ST level? Studies of larger cohorts at the national and international levels are urgently needed to explore these questions and broaden our understanding of this enigmatic organism.

## Data Availability

Molecular data generated in this project have been submitted to GenBank and are publicly available.

## Supplementary material

For supplementary material accompanying this paper visit http://doi.org/10.1017/S0950268823000596. click here to view supplementary material
