# Circulation and colonisation of Blastocystis subtypes in schoolchildren of various ethnicities in rural northern Thailand – Erratum

**DOI:** 10.1017/S095026882300078X

**Published:** 2023-06-02

**Authors:** Abby McCain, Lucsame Gruneck, Siam Popluechai, Anastasios D. Tsaousis, Eleni Gentekaki

**Affiliations:** 1 School of Science, Mae Fah Luang University, Chiang Rai, Thailand; 2 Gut Microbiome Research Group, Mae Fah Luang University, Chiang Rai, Thailand; 3 Laboratory of Molecular and Evolutionary Parasitology, RAPID Group, School of Biosciences, University of Kent, Canterbury, UK

When this article was originally published in Epidemiology & Infection it omitted to include Dr Eleni Gentekaki in the list of corresponding authors. This has been updated in the article.

The publisher apologises for this error.
