# Supplementary material for: Circulation and colonisation of Blastocystis subtypes in schoolchildren of various ethnicities in rural northern Thailand
Source: Epidemiol Infect. 2023 Apr 27;151:e77. doi: 10.1017/S0950268823000596 (PMC10204141; doi:10.1017/S0950268823000596)
Supplement: Supplementary file 1 [file S0950268823000596sup001.zip › S0950268823000596sup006.docx]

**Supplementary Table 1. Details of participants in this study and Blastocystis carriage at two timepoints 105 days apart**

| **Sample** | **Ethnicity** | **Delivery mode** | **Milk Source** | **BMI** | ***Blastocystis* +/- tp1** | ***Blastocystis***  **+/- tp2** |
| --- | --- | --- | --- | --- | --- | --- |
| MA202 | Akha | C | BM | SUW | * | + |
| MA203 | Akha | N | BM | LN | + | + |
| MA204 | Chinese | N | BM | LN | - | + |
| MA205 | Thai Yai | N | BM | OV | + | + |
| MA207 | Chinese | N | BM | LN | * | - |
| MA208 | Thai Yai | N | BM | LN | * | + |
| MA210 | Akha | N | BM | OB | + | + |
| MA211 | Chinese | N | BM | OV | * | + |
| MA212 | Thai | C | BM | UW | * | + |
| MA213 | Thai Yai | N | BM | LN | * | + |
| MA214 | Chinese | C | BM | OV | * | + |
| MA215 | Chinese | C | BM | LN | * | + |
| MA217 | Chinese | C | BM | OV | + | + |
| MA218 | Akha | N | BM | LN | * | + |
| MA221 | Chinese | C | BM | OB | * | + |
| MA222 | Thai Yai | N | BM | LN | * | + |
| MA224 | Thai | N | BM | LN | * | + |
| MA227 | Thai | N | BM | LN | + | + |
| MA228 | Akha | N | BM | LN | * | + |
| MA229 | Chinese | N | BM | LN | * | + |
| MA230 | Chinese | N | BM | OB | * | - |
| MA232 | Thai Yai | N | BM | LN | * | + |
| MA235 | Thai Yai | N | BM | LN | - | + |
| MA236 | Akha | N | BM | OB | + | + |
| MA237 | Thai | C | BM | LN | * | + |
| MA240 | Akha | N | BM | LN | * | - |
| MA241 | Akha | C | M | LN | + | + |
| MA242 | Thai Yai | C | BM | LN | * | + |
| MA243 | Thai Yai | N | BM | LN | * | - |
| MA244 | Akha | N | BM | LN | * | + |
| MA246 | Thai Yai | N | BM | LN | - | + |
| MA247 | Chinese | C | BM | OV | * | - |
| MA248 | Thai Yai | C | BM | OV | * | - |
| MA254 | Akha | N | M | LN | * | + |
| MA256 | Chinese | N | BM | OV | - | + |
| MA258 | Thai | N | BM | UW | * | + |
| MA259 | Lahu | C | M | LN | * | - |
| MA261 | Akha | N | BM | LN | * | - |
| MA263 | Thai | N | BM | LN | * | + |
| MA265 | Lahu | N | BM | LN | * | + |
| MA266 | Thai Yai | N | F | LN | * | + |
| MA267 | Thai | N | BM | LN | * | + |
| MA268 | Chinese | N | M | LN | * | + |
| MA270 | Thai Yai | C | BM | LN | - | + |
| MA271 | Thai Yai | C | F | LN | * | - |
| MA272 | Chinese | N | BM | OV | + | + |
| MA273 | Akha | C | BM | OV | * | + |
| MA276 | Thai | C | BM | LN | * | - |
| MA277 | Chinese | N | BM | OB | * | + |
| MA279 | Akha | C | BM | OV | * | + |
| MA280 | Thai | N | BM | OB | + | + |
| MA281 | Akha | C | BM | LN | * | + |
| MA282 | Chinese | C | M | OV | - | + |
| MA284 | Thai Yai | C | F | LN | * | + |
| MA286 | Thai | N | F | LN | + | + |
| MA287 | Thai | N | BM | LN | * | + |
| MA288 | Thai Yai | N | BM | LN | * | + |
| MA289 | Thai Yai | N | BM | LN | * | + |
| MA290 | Chinese | N | F | UW | * | + |
| MA291 | Chinese | N | F | OV | + | + |
| MA292 | Burmese | N | BM | OB | * | + |
| MA293 | Akha | N | BM | LN | * | + |
| MA295 | Thai Yai | N | BM | LN | * | + |
| MA296 | Lahu | N | BM | LN | + | + |
| MA299 | Akha | N | BM | LN | * | + |
| MA300 | Thai | C | BM | UW | - | + |
| MA301 | Thai | C | F | OB | * | + |
| MA302 | Thai | C | BM | OB | - | + |
| MA303 | Lahu | N | BM | LN | - | + |
| MA304 | Akha | N | BM | LN | * | + |
| MA306 | Akha | N | BM | LN | * | + |
| MA307 | Akha | N | BM | LN | + | + |
| MA308 | Thai Yai | N | BM | OV | * | + |
| MA309 | Akha | N | F | LN | + | + |
| MA311 | Akha | C | BM | LN | + | + |
| MA312 | Akha | C | F | LN | * | + |
| MA314 | Thai | C | F | OB | * | + |
| MA315 | Akha | N | BM | N/A | * | + |
| MA316 | Akha | N | F | OV | * | + |
| MA317 | Akha | C | BM | LN | * | + |
| MA318 | Akha | N | BM | OB | - | + |
| MA320 | Chinese | N | BM | LN | + | + |
| MA321 | Akha | C | BM | LN | * | + |
| MA323 | Chinese | C | BM | OB | - | + |
| MA325 | Chinese | C | F | OB | + | + |
| MA326 | Thai Yai | N | BM | LN | * | - |
| MA327 | Chinese | C | M | LN | + | + |
| MA328 | Thai Yai | C | M | LN | * | + |
| MA330 | Thai | C | BM | LN | * | + |
| MA331 | Akha | N | BM | OV | + | + |
| MA332 | Thai Yai | C | BM | LN | + | + |
| MA333 | Thai Yai | N | BM | LN | * | + |
| MA334 | Thai | C | BM | OB | + | + |
| MA336 | Lahu | C | BM | OB | - | + |
| MA337 | Thai Yai | N | BM | LN | * | + |
| MA340 | Akha | N | BM | LN | * | + |
| MA341 | Chinese | C | BM | LN | + | + |
| MA342 | Thai | N | BM | LN | * | + |
| MA343 | Thai | N | BM | OV | * | + |
| MA344 | Chinese | N | F | LN | + | + |
| MA345 | Chinese | N | F | LN | * | + |
| MA346 | Chinese | N | F | LN | * | + |
| MA348 | Thai Yai | C | F | OB | + | + |
| MA349 | Thai Yai | N | BM | OB | * | + |

BM= breast milk; C=Caesarean section; F=formula; LN=lean; M=mix breast milk+formula; N=natural birth; OB=obese; OV=overweight; SUW=severe underweight; UW=underweight *=no sequence available
