# Supplementary material for: Circulation and colonisation of Blastocystis subtypes in schoolchildren of various ethnicities in rural northern Thailand
Source: Epidemiol Infect. 2023 Apr 27;151:e77. doi: 10.1017/S0950268823000596 (PMC10204141; doi:10.1017/S0950268823000596)
Supplement: Supplementary file 1 [file S0950268823000596sup001.zip › S0950268823000596sup007.docx]

| **Sample** | **Time Point 1** | **Time Point 2** | **% Difference** |
| --- | --- | --- | --- |
| 203 | ST3/ST1 | ST3 | 2.79% |
| 205 | ST2 | ST2 | 0.00% |
| 210 | ST3 | ST3 | 0.18% |
| 217 | ST3 | ST3 | 0.79% |
| 227 | ST3 | ST3 | 0.18% |
| 241 | ST3 | ST3 | 0.00% |
| 280 | ST3 | ST3 | 0.00% |
| 286 | ST1 | ST1 | 1.35% |
| 291 | ST2 | ST2 | 0.30% |
| 296 | ST3 | ST3 | 2.97% |
| 309 | ST3 | ST3 | 0.74% |
| 311 | ST3 | ST3 | 0.00% |
| 320 | ST3 | ST3 | 0.00% |
| 325 | ST3 | ST3 | 0.18% |
| 327 | ST2 | ST2 | 0.00% |
| 331 | ST1 | ST1 | 0.00% |
| 334 | ST1 | ST1 | 1.44% |
| 344 | ST3 | ST1/ST3 | 0.72% |
