# Supplementary figures and images for: Circulation and colonisation of Blastocystis subtypes in schoolchildren of various ethnicities in rural northern Thailand
Source: Epidemiol Infect. 2023 Apr 27;151:e77. doi: 10.1017/S0950268823000596 (PMC10204141; doi:10.1017/S0950268823000596)

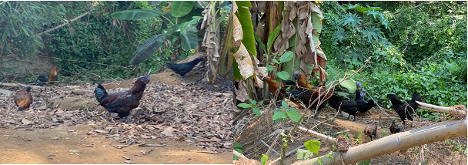

Supplement: Supplementary file 1 [file S0950268823000596sup001.zip › S0950268823000596sup001.png]

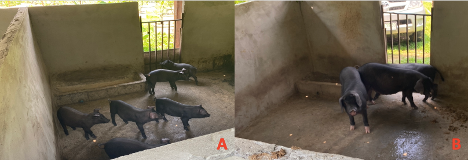

Supplement: Supplementary file 1 [file S0950268823000596sup001.zip › S0950268823000596sup002.png]

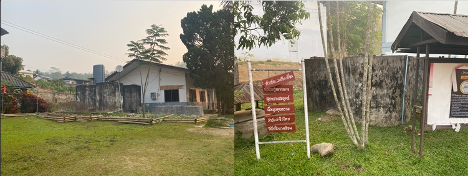

Supplement: Supplementary file 1 [file S0950268823000596sup001.zip › S0950268823000596sup003.png]

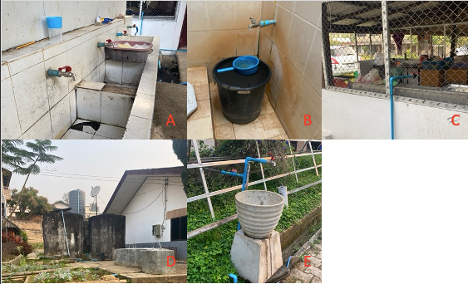

Supplement: Supplementary file 1 [file S0950268823000596sup001.zip › S0950268823000596sup004.png]

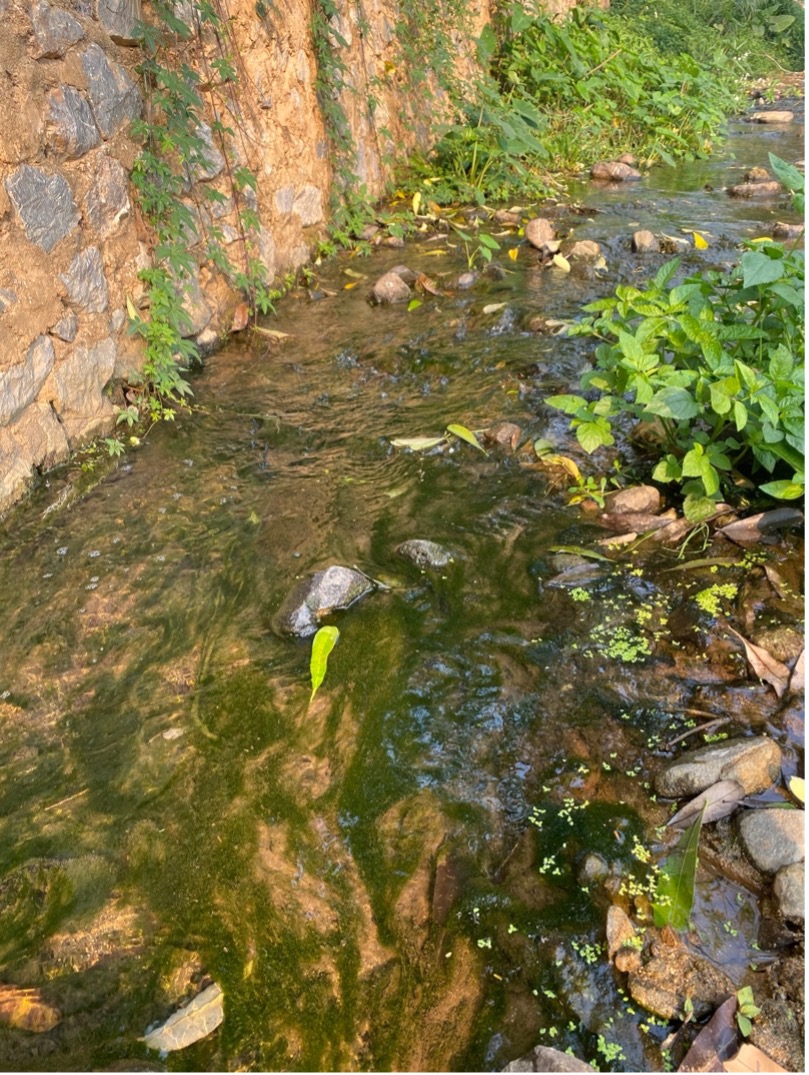

Supplement: Supplementary file 1 [file S0950268823000596sup001.zip › S0950268823000596sup005.jpg]
